# Signal-On Detection of Caspase-3 with Methylene Blue-Loaded Metal-Organic Frameworks as Signal Reporters

**DOI:** 10.3390/molecules29153700

**Published:** 2024-08-05

**Authors:** Yaliang Huang, Jiaqiang Wang, Yirui Xu, Jiwen Zhang, Ning Xia

**Affiliations:** 1 School of Pharmacy, Hunan University of Chinese Medicine, Changsha 410208, China; 2 College of Chemistry and Chemical Engineering, Anyang Normal University, Anyang 455000, China17838561822@163.com (J.Z.); 3 College of Chemistry and Chemical Engineering, Central South University, Changsha 410083, China

**Keywords:** caspase-3, cell apoptosis, metal–organic frameworks, electrochemical biosensor, biotinylation

## Abstract

In this work, we report on an electrochemical method for the signal-on detection of caspase-3 and the evaluation of apoptosis based on the biotinylation reaction and the signal amplification of methylene blue (MB)-loaded metal–organic frameworks (MOFs). Zr-based UiO-66-NH_2_ MOFs were used as the nanocarriers to load electroactive MB molecules. Recombinant hexahistidine (His_6_)-tagged streptavidin (rSA) was attached to the MOFs through the coordination interaction between the His_6_ tag in rSA and the metal ions on the surface of the MOFs. The acetylated peptide substrate Ac-GDEVDGGGPPPPC was immobilized on the gold electrode. In the presence of caspase-3, the peptide was specifically cleaved, leading to the release of the Ac-GDEVD sequence. A N-terminal amine group was generated and then biotinylated in the presence of biotin-NHS. Based on the strong interaction between rSA and biotin, rSA@MOF@MB was captured by the biotinylated peptide-modified electrode, producing a significantly amplified electrochemical signal. Caspase-3 was sensitively determined with a linear range from 0.1 to 25 pg/mL and a limit of detection down to 0.04 pg/mL. Further, the active caspase-3 in apoptosis inducer-treated HeLa cells was further quantified by this method. The proposed signal-on biosensor is compatible with the complex biological samples and shows great potential for apoptosis-related diagnosis and the screening of caspase-targeting drugs.

## 1. Introduction

Apoptosis is a highly regulated and complicated process of programed cell death for eliminating unwanted cells from tissues and maintaining normal physiological metabolism and tissue homeostasis [1]. The deregulation of apoptosis is closely related to the development of several severe diseases, such as cancers, autoimmune diseases, atherosclerosis, neurological disorder diseases, and so on [2,3,4]. Thus, the probing of cell apoptosis is essential for clinical investigation and apoptosis-targeted therapy. The progress of apoptosis can be indirectly monitored by measuring the activity of specific intracellular enzymes involved in the apoptotic pathway. Among them, caspases are a family of intracellular cysteine-dependent aspartate-specific proteases which play a critical role in regulating programmed cell death and apoptosis [5,6]. In particular, caspase-3, one of the most frequently activated cysteine proteases during the apoptosis process, has been considered a cellular biomarker of apoptosis [7]. In this aspect, the rapid, sensitive, and selective detection of caspase-3 and the evaluation of apoptosis can provide more important information for disease diagnosis and drug development.

Caspase-3 can specifically recognize and cleave the C terminus of the tetrapeptide motif Asp-Glu-Val-Asp (DEVD). Peptides containing the DEVD motif have been usually used as the substrates or probes for the evaluation of caspase-3 activity and apoptosis [8]. Currently, different analytical techniques have been reported for the detection of caspase-3 activity, including Western blotting [9], fluorescence [10,11,12], colorimetry [13,14,15], surface plasmon resonance spectroscopy [16,17], surface-enhanced Raman scattering spectroscopy [18,19,20], electrochemistry [21,22], electrochemiluminescence [23,24,25,26], and photoelectrochemistry [27,28]. The fluorescence assays are simple and sensitive for monitoring proteolytic events, but they require labor-intensive peptide substrates and specific instrumentation and show relatively low sensitivity [29]. Alternately, electrochemical methods have attracted considerable attention due to their excellent advantages of high sensitivity, rapid response, low cost, and simple operation [30,31]. Therein, diverse forms of voltammetry, such as square wave, differential pulse, linear sweep, and stripping, have been widely used as the electrochemical techniques for the assays of proteases [32,33]. Due to the weak redox activity of amino acids in peptides, the electrical signal generated by the proteolysis was too poor to achieve the sensitive measurement of proteases. For this reason, additional electroactive molecules, enzymes, and nanomaterials were employed as the signal reporters to label the peptide substrates [34,35]. The cleavage of a sequence-specific peptide substrate into two short segments by proteases can cause the release of signal reporters from the electrode surface, leading to the conversion of the proteolysis event into an electrochemical signal. Typically, ferrocene, *p*-nitroaniline, and *p*-methoxyaniline have been conjugated with peptide substrates for the electrochemical detection of caspase-3 activity and cell apoptosis, respectively [36,37,38]. To further improve sensitivity, Rashidi et al. used horseradish peroxidase-loaded magnetic beads to label the peptide substrate and realized the electrochemical detection of caspase-3 activity during hematopoietic stem cell differentiation [39]. However, the immobilization of a labeled peptide on the solid surface may prevent the interaction between peptide and the active center of proteases due to steric hindrance, thus decreasing enzymatic efficiency [12,40]. To decrease the effect, biotin-labeled peptides could be used as the caspase-3 substrates and streptavidin (SA)-modified materials could be employed to recognize the biotin labels remaining on the electrode surface, such as SA-conjugated alkaline phosphatase [41] and quantum dots-functionalized carbon nanotubes [42]. Additionally, Liu et al. reported an impedimetric biosensor for the determination of caspase-3 activity and cell apoptosis with the self-assembled biotin–phenylalanine network as the signal amplifier [43]. Chen et al. synthesized SA-modified Co_3_O_4_–Au polyhedra as a signal enhancer to sensitively detect caspase-3 [44]. However, most of these methods are always performed in a signal-off mode, resulting in low signal-to-background ratios.

**Scheme 1:**
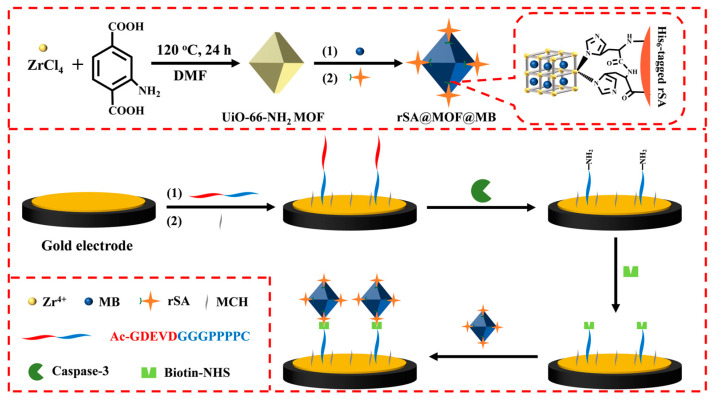
A schematic illustration of the preparation of rSA@MOF@MB and the principle for the electrochemical detection of caspase-3.

Alternatively, protease-catalyzed protein/peptide hydrolysis can produce new N termini, and the exposed amino group can serve as a reactive site to be specifically labeled by specific molecules or nanomaterials [45,46,47]. For instance, biotin-labeled 2-pydidinecarboxyaldehyde was used to selectively react with protein N termini under mild pH and temperature conditions [48,49]. Chen et al. reported an electrochemical method for caspase-3 detection by using *p*-sulfonatocalix [6]arenes sodium-modified graphene oxide to recognize the exposed amine group for the further capture of electroactive methylene blue (MB) via the host–guest interaction [50]. He et al. used citrate-capped silver nanoparticles as electroactive tags to label the generated amine group [51]. However, these nanomaterials are prone to aggregate because of different factors, such as high salt concentration, thiol molecules in samples, or weak intermolecular π–π and Van der Waals interactions. Nanocontainers including liposomes, polymers, and hollow or mesoporous structures are able to entrap small molecules for drug delivery, theranostics, biomedicine, and nanoreactors [52,53,54]. As an important family of porous crystalline materials, metal–organic frameworks (MOFs) are composed by metal ions/clusters and organic ligands via the coordination interaction [55,56]. Recently, MOFs have been widely used in the development of electrochemical biosensors due to their inherent properties of tunable porous structure, large specific surface area, abundant functional sites, good chemical stability, and excellent biocompatibility [57,58]. The highly ordered pores and large specific surface area endow MOFs with the capability to serve as nanocarriers for signal reporters, such as dyes, redox molecules, enzymes, and nanoparticles. In addition, the abundant coordinatively unsaturated metal sites on their external surface can facilitate the self-assembly of hexahistidine (His_6_)-tagged recombinant biorecognition elements onto the MOF surface via the coordination interactions between the histidine residues in the His_6_ tags and the metal ions/clusters [59]. In this work, a signal-on electrochemical biosensor for caspase-3 detection was developed by integrating the biotinylation of the exposed amine group with recombinant SA (rSA) and MB-modified MOFs (Scheme 1). Zr-based UiO-66-NH_2_ MOFs were used as the nanocarriers to load redox-active MB and His_6_-tagged rSA. After catalyzed cleavage and subsequent biotinylation reactions, rSA@MOF@MB was captured by the biotinylated peptide on the electrode. A large number of MB molecules in MOF could generate an amplified electrochemical signal for sensitive detection of caspase-3 and the monitoring of cell apoptosis with a low limit of detection. The signal-on strategy could be helpful for the diagnosis and prognosis of apoptosis-related diseases and the development of novel protease biosensors.

## 2. Results and Discussion

### 2.1. Principle of the Method

It has been demonstrated that a spacer module, such as synthetic alkane chains, poly(ethylene glycol) (PEG), and polyproline, is the key to overcoming or reducing the steric hindrance effect between the solid interface and the target [60]. Among them, polyproline, a neutral, compact, and rigid linker, exhibits both good water solubility and hydrophobicity due to the lack of intramolecular hydrogen bonds. More importantly, polyproline can be readily inserted into the peptide during the synthesis process and pattern tight self-assembled monolayers on the solid surface to eliminate non-specific adsorption [61]. Based on this fact, the peptide substrate was designed by connecting the identified DEVD cleavage sequence and the C-terminal cysteine with the linker polyproline (PPPP). The N terminus of the substrate peptide was blocked by an acetyl group, which could not react with the biotinylation reagent biotin-NHS. In the assay, the peptide with a cysteine residue at the C terminus was immobilized on the gold electrode via the robust interaction between Au and the thiol group in cysteine. The acetylated peptide could not react with biotin-NHS due to the absence of a free amino group, and rSA@MOF@MB could not be immobilized on the surface of the peptide-modified electrode. However, in the presence of caspase-3, the cleavage of the peptide by caspase-3 led to the release of acetylated pieces. The exposed amine group could be functionalized with the biotin group in the presence of biotin-NHS. Then, rSA@MOF@MB was captured by the biotinylated peptide-modified electrode surface via the specific binding between rSA and biotin. The differential pulse voltammetry (DPV) current is proportional to the amount of rSA@MOF@MB on the sensing interface, corresponding to the biotinylated peptide, which is dependent on the level and activity of caspase-3.

### 2.2. Characterization of MOFs

**Figure 1:**
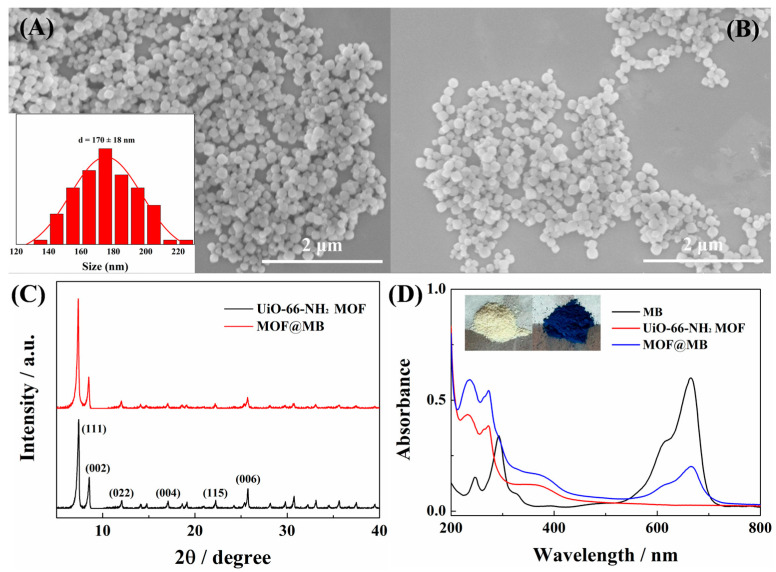
SEM images of (**A**) UiO-66-NH_2_ MOF and (**B**) MOF@MB. (**C**) XRD patterns of UiO-66-NH_2_ MOF (black curve) and MOF@MB (red curve). (**D**) UV–vis absorption spectra of MB (black curve), UiO-66-NH_2_ MOF (red curve), and MOF@MB (blue curve). The inset in panel A shows the size distribution of the UiO-66-NH_2_ MOF.

**Figure 2:**
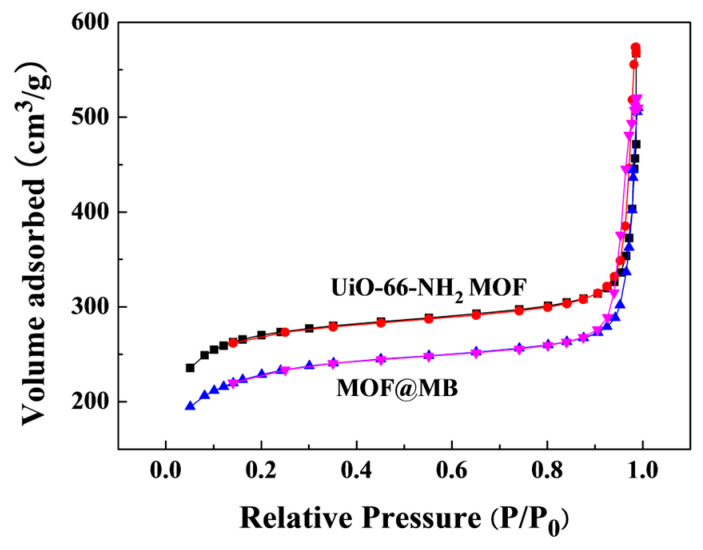
BET analysis of UiO-66-NH_2_ MOF and MOF@MB.

UiO-66-NH_2_ MOFs were prepared by a conventional solvothermal method and used as the nanocarriers for the accumulation of abundant redox MB molecules due to their high porosity and large surface area [62,63]. The morphologies and structures of UiO-66-NH_2_ MOF and MOF@MB were characterized by SEM and XRD analyses. As presented in Figure 1A, the as-prepared UiO-66-NH_2_ MOFs showed a typical octahedron structure with an average size of approximately 170 nm. After the encapsulation of MB, the resulting MOF@MB exhibited similar morphology (Figure 1B). The XRD pattern was further used to characterize the crystal structure of UiO-66-NH_2_ and MOF@MB. The characteristic peaks are shown in Figure 1C. The results demonstrate that the synthesized UiO-66-NH_2_ MOF had good morphology, good purity, and high crystallinity, and the encapsulation of MB did not change the morphology and structure of the UiO-66-NH_2_ MOFs. In addition, the UV–vis spectra of the synthesized MOF materials were collected to confirm the successful encapsulation of the MB molecules. As shown in Figure 1D, there were the absorption peaks at 237 nm and 274 nm in the spectrum of UiO-66-NH_2_ (red curve) due to the transition of the conjugated π electron of the ligand to the Zr center [64]. After the encapsulation of MB molecules, a new absorption peak at 665 nm in the spectrum of UiO-66-NH_2_@MB was observed (blue curve), which could be attributed to the n-π* absorption of the MB molecules (black curve) [65]. Further, the color of the MOF powder changed from faint yellow to deep blue (see the inset). In addition, the changes in the BET surface area and pore volume of the MOFs after the encapsulation of MB were investigated. It was found that the BET surface area and pore volume changed from 843 m^2^/g and 0.878 cm^3^/g to 725 m^2^/g and 0.788 cm^3^/g, respectively (Figure 2). The above results demonstrate that the MB molecules were successfully entrapped into the UiO-66-NH_2_ MOFs. To prove that rSA was attached onto the surface of MOF@MB, the change in the Zeta potential of the MOFs was determined. It was found that the Zeta potential of the MOFs changed from 32.8 to 36.1 mV after the entrapment of MB, and changed to 35.2 mV after the attachment of rSA.

### 2.3. Feasibility

**Figure 3:**
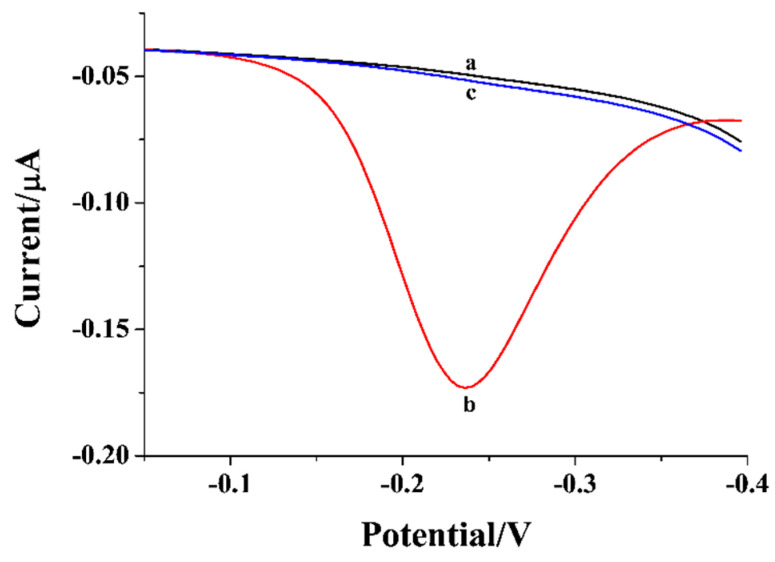
DPV responses corresponding to the peptide-modified sensing electrode after treatment by biotin-NHS + rSA@MOF@MB (**a**), caspase-3 + biotin-NHS + rSA@MOF@MB (**b**), and caspase-3 + rSA@MOF@MB (**c**).

Figure 3 shows the DPV responses of the peptide-modified sensing electrode before and after treatment by caspase-3 and follow-up incubation with biotin-NHS and rSA@MOF@MB. In the absence of caspase-3, no voltammetric signal was observed, even when the electrode was incubated with biotin-NHS and rSA@MOF@MB (curve a). The results indicate that the acetylated peptide did not allow for the attachment of biotin-NHS and rSA@MOF@MB on the electrode surface. However, an obviously increased signal was observed when the electrode was treated by caspase-3, biotin-NHS, and rSA@MOF@MB (curve b). This implied that the peptide was cleaved by caspase-3 and the exposed free amino group could be biotinylated for the attachment of rSA@MOF@MB on the electrode surface. Therefore, numerous MB molecules in the MOFs generated a high electrochemical signal. A control experiment was conducted by incubating the caspase-3-treated electrode with rSA@MOF@MB directly, and the electrochemical signal was nearly equal to the blank (curve c), demonstrating that biotinylation is required for the capture of rSA@MOF@MB. These results suggest that the turn-on signal from the captured rSA@MOF@MB was dependent upon the cleavage of peptide by caspase-3 and the biotinylation of the exposed amine group.

### 2.4. Optimization of Detection Conditions

**Figure 4:**
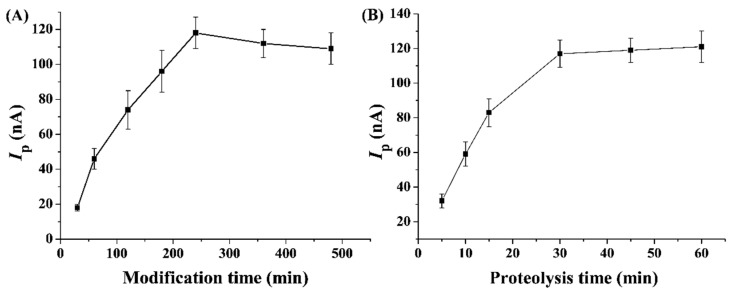
The optimized times for peptide modification (**A**) and proteolysis (**B**).

To achieve the best detection performance of the method, two important factors were optimized. Appropriate coverage is beneficial for the cleavage of the substrate peptide. Accordingly, the incubation time for peptide immobilization was first optimized. As shown in Figure 4A, the electrochemical signal increased with the increase in modification time. The current reached the maximum at the modification time of 240 min and began to decline slightly, which is probably due to the steric hindrance from the peptide with high density on the electrode. Thus, 240 min was used as the ideal time for the immobilization of the peptide. We also found that the electrochemical signal was dependent on the proteolysis time (Figure 4B). The current increased and began to level off after 30 min of proteolysis. Therefore, 30 min was selected as the casapse-3 proteolysis time in this study.

### 2.5. Analytical Performance of Method

**Figure 5:**
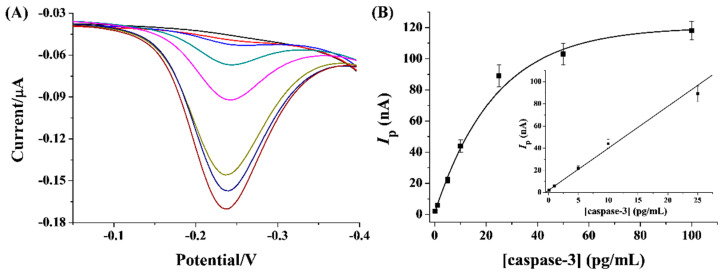
(**A**) DPV responses of the electrochemical biosensor at different concentrations of caspase-3 (from top to bottom: 0, 0.1, 1, 5, 10, 25, 50, and 100 pg/mL). (**B**) The relationship between peak current and caspase-3 concentration. The inset shows the linear portion of the fitting curve.

To ensure the applicability of the method, the prepared biosensors with rSA@MOF@MB as the signal reporters were used to detect different concentrations of caspase-3 under the optimized conditions. As shown in Figure 5A, the DPV signal increased with the increment in caspase-3 concentration from 0 to 100 pg/mL. Notably, the electrochemical signal was negligible without the incubation of caspase-3, demonstrating that the background current and the non-specific adsorption were negligible. There was a good linear relationship between the current and the concentration of caspase-3 in the range from 0.1 to 25 pg/mL. The limit of detection (LOD) was calculated to be 0.04 pg/mL, which was comparable to or even lower than that of other nanomaterial or enzyme-based signal reporters for signal amplification (Table 1). It is noteworthy that the peptide used in this work can ensure high-efficiency proteolysis and high antifouling ability, and the easy preparation and modification of rSA@MOF@MB signal tags simplify the experimental complexity.

### 2.6. Selectivity of Method and Evaluation of Inhibition Viability

**Figure 6:**
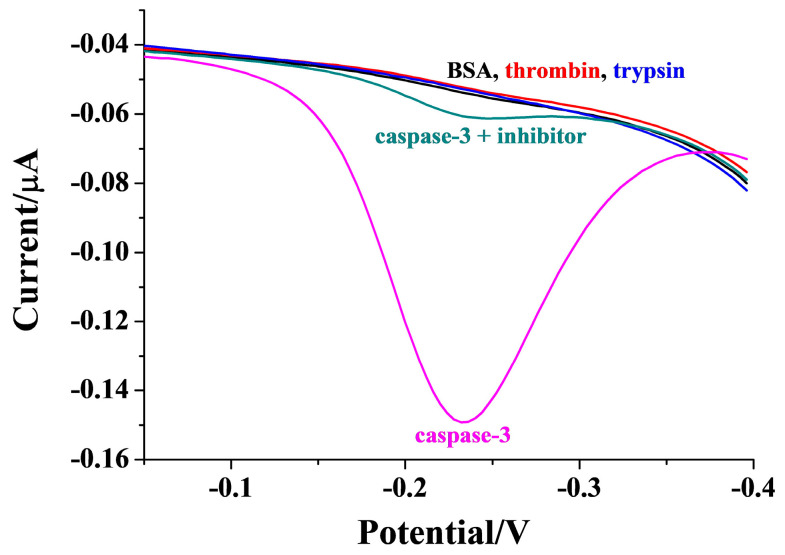
DPV responses of the electrochemical biosensor for the detection of different samples. The concentrations of caspase-3, inhibitor, BSA, thrombin, and trypsin were 25 pg/mL, 50 pM, 10 ng/mL, 1 ng/mL, and 1 ng/mL, respectively.

To evaluate the specificity of the method for caspase-3 detection, the biosensor was incubated with BSA and two proteases (thrombin and trypsin) as interfering substances. Under the same experimental conditions, the DPV currents for the assays of these samples were collected. As illustrated in Figure 6, the currents for the non-targets were close to that of the blank control. The result suggests that the proposed method had an excellent selectivity toward caspase-3. In addition, we found that the current for the detection of caspase-3 was 92% of the original value after the storage of rSA@MOF@MB at 4 °C for three months, indicating that the signal reporters exhibited good stability.

**Figure 7:**
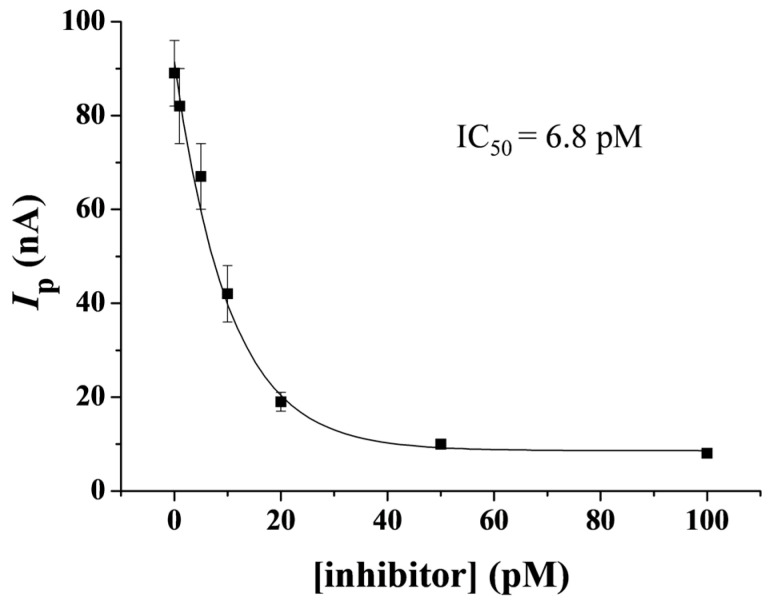
The relationship between peak current and inhibitor concentration for the assay of 25 pg/mL caspase-3.

To demonstrate that the signal change was attributed to the enzymatic activity of caspase-3, DEVD-FMK was used to inhibit the activity of caspase-3. As a result, the current in the presence of DEVD-FMK was remarkably lower than that without the addition of inhibitor. Moreover, the responses exhibited a dependence on the inhibitor concentration (Figure 7), suggesting that the signal change arose from the catalytic activity of caspase-3. From the corresponding relationship between the inhibition viability of DEVD-FMK for caspase-3 and the inhibitor concentration, the half-maximum inhibition value (IC_50_) was found to be about 6.8 pM. The value is in good agreement with that determined by other methods [8,43], indicating that the biosensor has the potential to screen potential inhibitors as therapeutic drugs.

### 2.7. Assays of Caspase-3 in HeLa Cells

**Figure 8:**
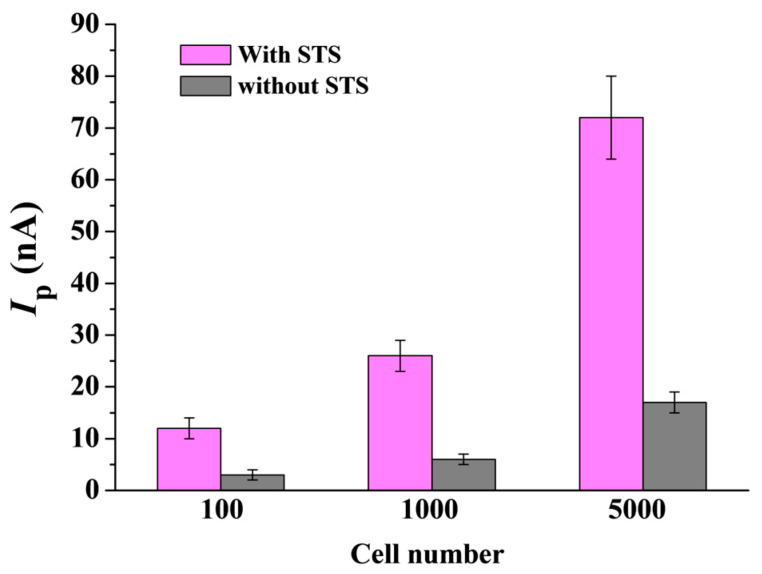
The relationship between peak current and cell number for the assays of caspase-3 in HeLa cells with and without treatment by STS.

To evaluate the analytical reliability and applicability, the developed biosensor was employed to monitor drug-induced cell apoptosis. In this work, HeLa cells were used as the model cell type for apoptosis and treated with STS, a commonly used apoptosis inducer. At the same time, a control group was prepared with the same cell concentration without incubation with STS. As presented in Figure 8, a small current was observed in the control group, indicating that caspase-3 was inactive in HeLa cells. However, a significant increase in the current was observed when the cells were treated with STS, demonstrating that caspase-3 in the apoptotic cells was activated to efficiently cleave the peptide. Moreover, the current increased significantly with the increase in the number of apoptotic HeLa cells, which further confirmed the practicability and effectiveness of the designed biosensor for apoptosis evaluation.

## 3. Materials and Methods

### 3.1. Chemicals and Reagents

Zirconium chloride (ZrCl_4_), MB, and benzoic acid were supplied by Titan Co. Ltd. (Shanghai, China). Aminoterephthalic acid (NH_2_-BDC), 6-mercapto-1-hexanol (MCH), and tris(2-carboxyethyl)phosphine hydrochloride (TCEP) were provided by Sigma-Aldrich (Shanghai, China). Caspase-3 was purchased from New England BioLabs (Ipswich, MA, USA). Glucose oxidase, trypsin, thrombin, *β*-secretase, and Dulbecco’s modified Eagle’s medium (DMEM) were provided by Sangon Biotech. Co., Ltd. (Shanghai, China). Staurosporine (STS) and other reagents were obtained from Aladdin Company (Shanghai, China). The peptide with the sequence Ac-GDEVDGGGPPPPC was synthesized and purified by Synpeptides Co., Ltd. (Shanghai, China). All regents in the experiment were of analytical grade, and all the stock solutions were prepared with ultrapure water (18.2 MΩ/cm) from a Millipore water purification system.

### 3.2. Apparatus

Electrochemical measurements were conducted on a CHI 660E electrochemical workstation (Shanghai, China). The three-electrode system consisted of a gold (Au) electrode as the working electrode, a Ag/AgCl electrode (immersed in saturated KCl solution) as the reference electrode, and a Pt wire as the auxiliary electrode. The UV–vis spectra were recorded on a spectrophotometer (Cary 60; Agilent, Santa Clara, CA, USA). The morphological analysis was performed with scanning electron microscopy (SEM) equipment. The phase constituents of the MOFs were characterized by an Ultima III X-ray diffraction (XRD) apparatus. The N_2_ adsorption–desorption experiments were conducted at 77 K on a Micromeritics Gemini VII surface area and porosity instrument (Norcross, GA, USA). The Zeta potential of the MOFs was determined by a Nano ZS90 particle size analyzer.

### 3.3. Preparation of rSA@MOF@MB

The UiO-66-NH_2_ MOFs were prepared according to the previously reported work [62]. In detail, ZrCl_4_ (0.120 g), NH_2_-BDC (0.110 g), and benzoic acid (1.9 g) were dissolved in 10 mL of DMF, and the mixture was treated with sonication for 10 min. Then, the solution was transferred into a Teflon-lined autoclave and placed in an oven at 120 °C for 24 h. After that, the solution was cooled to room temperature, and the product was collected by centrifugation at 8000 rpm for 10 min and then washed with DMF and ethanol three times, respectively. Finally, the precipitates were dried at 60 °C under vacuum overnight.

The UiO-66-NH_2_ MOFs were used to carry MB molecules based on the reported literature [63]. Briefly, the as-synthesized pale yellow UiO-66-NH_2_ MOFs (25 mg) were dispersed in 25 mL of water with the aid of sonication for 15 min. Next, MB (2.5 mg) was added to the solution, which was stirred for 24 h at room temperature. The final solid was collected by centrifugation at 8000 rpm for 10 min and washed with water thoroughly. The prepared MOF@MB samples were dried for further use.

The assembly of rSA on the MOFs was achieved based on metal–His_6_ coordination interactions [59,67]. MOF@MB (0.1 mg/mL) was dispersed in the phosphate buffer (10 mM, pH 7.4) and then mixed with the rSA solution (50 μg/mL). After mild shaking at room temperature for 1 h, the suspension was centrifuged at 8000 rpm for 10 min, and the solid was washed with the buffer several times. The obtained rSA@MOF@MB was stored at 4 °C for further use.

### 3.4. Electrode Modification

The Au electrode was treated by a fresh piranha solution consisting of H_2_SO_4_ and 30% H_2_O_2_ in a volume ratio of 3:1 for approximately 10 min. After being rinsed thoroughly with ultrapure water, the electrode was polished with 0.05 µm alumina slurry and washed ultrasonically in 50% ethanol for a few seconds. After that, the electrode was scanned in 0.5 M H_2_SO_4_ for 10 circles with a scan rate of 0.1 V/s and a potential range from –0.3 to 1.6 V. After having been cleaned with ultrapure water and dried in N_2_ atmosphere, the Au electrode was incubated with the phosphate buffer (10 mM, pH 7.4) containing 10 μM peptide and 0.5 mM TCEP at 4 °C and washed with the buffer. Then, the peptide-modified electrode was immersed into a 1 mM MCH solution for 1 h to block the unreacted sites and reduce non-specific adsorption. The resulting sensing electrode was washed with ethanol and water for further use.

### 3.5. Electrochemical Detection of Caspase-3

The peptide-modified sensing electrode was incubated with HEPES buffer (10 mM, pH 7.4) containing a given concentration of caspase-3 at room temperature for a given time. After having been washed with water, the electrode was immersed into the DMF solution containing 0.1 mM biotin-NHS. After reacting for 15 min, the electrode was washed and further incubated with 0.01 mg/mL rSA@MOF@MB suspension for 10 min; then, the electrode was washed thoroughly and subjected to electrochemical measurement in the Tris-HCl buffer solution (20 mM, pH 7.4) containing 0.1 M NaCl. For inhibitor evaluation, the experiment was performed by using the same procedure, except that caspase-3 was replaced with a mixture of DEVD-FMK and caspase-3.

### 3.6. Cell Culture and Apoptosis Assays

The procedures for cell culture and the preparation of cell lysates followed our previous report [43]. Briefly, HeLa cells were incubated in Dulbecco’s modified Eagle’s medium (DMEM) supplemented with 10% fetal calf serum. They were then cultured at 37 °C in a humidified atmosphere with 5% CO_2_ for 1 day. The tested inducer STS was added to induce apoptosis. After incubation for 8 h, the cells were washed and lysed with the ice-cold lysis buffer. Next, 0.5 mL of the lysate was transferred to a centrifuge tube and centrifuged at 12,000 rpm for 10 min at 4 °C. The supernatant was collected and diluted with assay buffer for the assays. Caspase-3 in the diluted lysate was analyzed by following the aforementioned procedure.

## 4. Conclusions

In summary, we developed an electrochemical biosensor for the signal-on detection of caspase-3 and the evaluation of cell apoptosis based on the biotinylation reaction and multifunctional MOFs. UiO-66-NH_2_ MOFs served as the nanocarriers to load electroactive MB molecules and as the platforms to anchor rSA proteins through the interactions between His_6_ tags and metal ions. The exposed amino group generated from caspase-3 proteolysis was first biotinylated and further reacted with rSA@MOF@MB via the biotin–rSA interactions. The developed electrochemical biosensor showed a good linear range and a low LOD for caspase-3 detection. Furthermore, this method was used to evaluate inhibition efficiency and drug-induced cell apoptosis, providing a new signal amplification strategy for protease assays in clinical applications.

## Figures and Tables

**Table 1:** Comparison between proposed method and other electrochemical biosensors for detection of caspase-3.

Signal Labels	Linear Range	LOD	Ref.
MB-carried GO	0.1–100 pg/mL	0.06 pg/mL	[45]
AgNPs	0.5–50 pg/mL	0.1 pg/mL	[51]
HRP-modified magnetic bead	100 pM–1 nM	100 pM	[46]
CB[8]-AgNPs	1–10 ng/mL	24.62 pg/mL	[47]
MB-*p*SC6-GO	10–100 pg/mL	0.0167 pg/mL	[50]
ATCUN-Cu(II) complex	0.5 pg/mL–2 ng/mL	0.2 pg/mL	[8]
biotin-Phe nanoparticle	1–125 pg/mL	1 pg/mL	[43]
AgNPs-modified HeLa cells	0.02–0.2 U/mL	0.02 U/mL	[66]
rSA@MOF@MB	0.1–25 pg/mL	0.04 pg/mL	This work

Abbreviation: SA, streptavidin; MB, methylene blue; AgNPs, silver nanoparticles; HRP, horseradish peroxidase; CB[8], cucurbit[8]uril; *p*SC_6_, *p*-Sulfonatocalix[6]arenes sodium; ATCUN, amino terminal Cu(II) and Ni(II)-binding motif; Phe, phenylalanine.

## Data Availability

The data presented in this study are available on request from the corresponding author due to privacy.
